# Exploring one-year mental health outcomes in a cohort of severe COVID-19 survivors: a focus on depressive and anxiety symptoms

**DOI:** 10.1192/j.eurpsy.2025.2054

**Published:** 2025-08-26

**Authors:** H. Salgado, S. Martins, L. Fontes, A. R. Ferreira, I. Coimbra, A. Braga, J. A. Paiva, L. Fernandes

**Affiliations:** 1 Psychiatry Service, Unidade Local de Saúde São João, Porto.; 2 Department of Clinical Neurosciences and Mental Health, Faculty of Medicine, University of Porto.; 3 Center for Health Technology and Services Research (CINTESIS@RISE), Faculty of Medicine, University of Porto.; 4 Intensive Care Medicine Department, Unidade Local de Saúde São João, Porto; 5 Department of Medicine, Faculty of Medicine, University of Porto, Porto, Portugal

## Abstract

**Introduction:**

The COVID-19 pandemic has evolved into substantial mortality and morbidity worldwide, with 5-8% of patients requiring intensive care. Years after the emergence of COVID-19, there is a heightened interest about the Long COVID, a heterogeneous condition characterized by persistent or emergent symptoms that last for weeks or months after patient’s recovery from acute infection, with a negative impact on physical and mental health. Research indicates that depression and anxiety often co-occur and may serve as predictors for Long COVID. Given the scale of the pandemic, even a small proportion of patients with long-lasting symptoms will create a significant health burden. Therefore, gathering data on the long-term evolution and outcomes of these patients is of utmost importance.

**Objectives:**

To identify depressive and anxiety symptoms in severe COVID-19 survivors 1-year after discharge and to analyse their association with sex, age and fear of recurrence and sequelae of COVID-19.

**Methods:**

The cohort analysed is part of MAPA research project and includes adult patients admitted due to COVID-19, in an Intensive Care Medicine Department of a University Hospital in Portugal. Participants were evaluated 1-year after discharge to home with a comprehensive protocol, which included Patient Health Questionnaire (PHQ-9; depressive symptoms), General Anxiety Disorder scale (GAD-7; anxiety symptoms) and a brief questionnaire about fear of recurrence and sequelae of COVID-19.

**Results:**

The final sample (n=159) had a mean age of 62.2 years and mostly (69%) was male. About 19% and 21% of survivors scored for depressive and anxiety symptoms, respectively. Both symptoms were significantly more prevalent among younger participants (p=0.031; p<0.001) and were associated with fear of recurrence (p=0.002; p=0.009) and sequelae (p=0.001; p<0.001) of COVID-19 1-year after discharge.

**Conclusions:**

Overall, there is a significant prevalence of anxiety and depressive symptoms among COVID-19 survivors 1-year after discharge. These findings emphasize the need for greater attention to these symptoms in this population, since its recognition and treatment can improve quality of life and reduce symptoms in the long term, especially in younger patients.

This work was supported by National Funds through FCT - Fundação para a Ciência e a Tecnologia,I.P., within CINTESIS, R&D Unit (reference UIDP/4255/2020)

**Disclosure of Interest:**

None Declared

